# The pore structure and water absorption in Portland/slag blended hardened cement paste determined by synchrotron X-ray microtomography and neutron radiography

**DOI:** 10.1039/d3ra06489a

**Published:** 2024-02-01

**Authors:** James E. Vigor, Dale P. Prentice, Xianghui Xiao, Susan A. Bernal, John L. Provis

**Affiliations:** a School of Civil Engineering, University of Leeds Woodhouse Lane Leeds LS2 9JT UK s.a.bernallopez@leeds.ac.uk; b Department of Materials Science and Engineering, The University of Sheffield Sheffield S1 3JD UK; c Department of Civil and Environmental Engineering, Imperial College London London SW7 2BX UK; d Civil and Environmental Engineering Department, University of California Los Angeles CA 90095 USA; e Argonne National Laboratory 9700 S. Cass Avenue Lemont IL 60439 USA; f Brookhaven National Laboratory 98 Rochester Road Upton NY 11973 USA; g Paul Scherrer Institut Forschungsstrasse 111 5232 Villigen PSI Switzerland john.provis@psi.ch

## Abstract

The pore structures of hardened Portland/slag cement pastes (>75 wt% slag content), and the initial capillary absorption of moisture through these pores, were monitored using *ex situ* synchrotron X-ray computerised microtomography and *in situ* quantitative neutron radiography. The pore structure becomes more constricted as the cement hydrates and its microstructure develops. This mechanism was effective even at a slag content as high as 90 wt% in the cementitious blend, where the lowest total porosity and a significant pore refinement were identified at extended curing ages (360 d). By combining this information with neutron radiographic imaging, and directly quantifying both depth and mass of water uptake, it was observed that 90 wt% slag cement outperformed the 75 wt% slag blend at 90 days in terms of resistance to capillary water uptake, although the higher-slag blend had not yet developed such a refined microstructure at 28 days of curing. The assumptions associated with the “sharp front model” for water ingress do not hold true for highly substituted slag cement pastes. Testing transport properties at 28 days may not give a true indication of the performance of these materials in service in the long term.

## Abstract 2

The development of the pore structures of hardened Portland/slag cement pastes for sample ages up to 1 year, and the absorption of moisture through these pores, are monitored and quantified using X-ray and neutron techniques.

## Graphical abstract

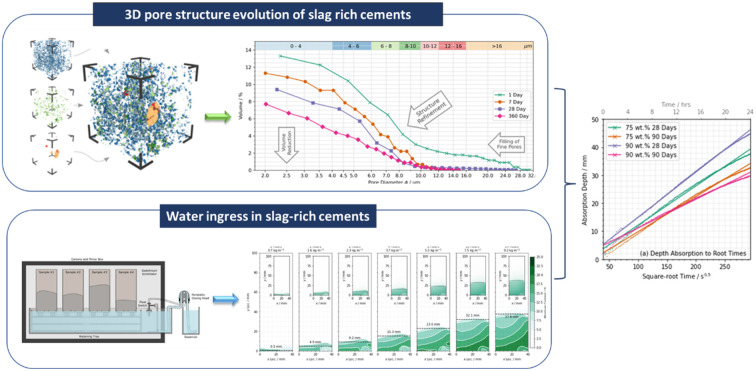

## 1. Introduction

The long-term performance of hardened cementitious materials is strongly dependent on physical and chemical interactions with their in-service environment, and in particular the ability to prevent potentially aggressive chemical species from entering the cementitious microstructure. The pore structure in cementitious materials results mainly from the initially water-filled spaces that are present in between irregularly-shaped anhydrous clinker grains, because a water content higher than can be fully chemically combined by hydration is usually required to enable concretes (and pastes and mortars) to be properly mixed and flowable.^1^ This causes the formation of micron to sub-micron capillary pathways in the solidified product. As the material reacts, in-filling of the pore space occurs due to the formation of voluminous hydrate products, which evolve from particle surfaces and precipitate into the initially water-filled pore space, and this can be monitored and imaged in three dimensions by tomographic techniques.^2^

In unsaturated hardened cement pastes and concretes, the ingress of moisture is primarily driven by capillary absorption, and may enable chemical and physical interactions to undermine the integrity of the material. Water entering the pore structure may carry chloride that places steel reinforcement at risk of corrosion,^3^ or may mediate the process of cement carbonation through CO_2_ ingress,^4^ and these processes may either be hindered or exacerbated by the presence of high volumes of supplementary cementitious materials (SCMs).^5^ Moisture movement plays an essential part in determining the rate and extent of these processes, and so must be understood in more detail to ensure material design for optimal performance.

Numerous test methods have been devised and subsequently standardised to evaluate geometric properties of the pore space within the solidified material, such as measurement of the pore size distribution using mercury intrusion porosimetry (MIP)^6^ and the measurement of the total volume of pore space using gravimetry.^7^ Standardised tests to measure flow properties such as the initial capillary absorption that occurs as dry cement samples, concretes, or porous materials are introduced to water have also been devised.^8^ However, those methods offer only low-resolution results without directly connecting the pore structure to the infiltration depth or the geometry of the wetting front. Debate about how best to determine transport properties of cementitious materials is ongoing in the literature.^9,10^ Evidence has also been presented regarding the effect of common sample preconditioning methods (*e.g.* high temperature drying), particularly if microcracking occurs.^11,12^ It is therefore uncertain whether some test methods are truly an accurate representation of what might be occurring within a cement-based material under realistic service conditions.

As an alternative method that can also be applied to the analysis of moisture transport in cements, neutron radiography (NR) enables dynamic investigation of processes involving hydrogen-rich compounds such as water, which interact strongly with neutrons, moving within samples that are otherwise of low neutron cross-section. Calibration of the neutron attenuation of the sample to the attenuation of a sample of water with a known path length enables moisture quantification, and therefore NR (particularly quantitative NR, denoted QNR) has become more widespread in research into porous media^13^ including applications in cementitious materials research.^14^ NR has been used to monitor the drying process in mortars^15^ and kaolinitic clays,^16^ rapid processes such as the release of water from porous aggregates,^17^ and the autogenous healing of concretes blended with superabsorbent polymers.^18,19^ Moreover, QNR has been validated as a reasonably accurate measurement of moisture content in cementitious materials following the appropriate removal of scattering artifacts.^20^

The processes occurring within a macroscale hydrated cement paste sample which can be observed using NR are linked to the micron-to sub-micron-scale properties of the cement pore structure, which can also be probed by cognate techniques including high-resolution X-ray microtomographic imaging (μCT).^21,22^ Due to the ability of X-ray microtomography to provide data in three-dimensions on this length-scale of around a micron, this method has previously been applied to quantify pore structure tortuosity in cementitious materials,^23–25^ and the distribution of pore sizes.^26,27^ Due to the unique insight that can be gained when applying NR and μCT, these two techniques have been combined to provide new insight into the characteristics of construction materials.^28^ The observations of pore structure from μCT experiments can also be linked to the permeability of the bulk material.^29^ The tortuosity of a pore network is a measurement of the free path complexity, and has been related to the mass transport properties of concrete.^30,31^ As the pore structure increases in complexity, as hydrates fill and expand from anhydrous materials into the inter-particle space, there is a reduction of the free-path length of the pore space, and the tortuosity increases. Due to the difficulty of directly measuring tortuosity using standard laboratory apparatus, this has more often been determined using the random walk method on microtomographic data,^24^ although the finite resolution of tomographic imaging does present challenges in interpretation of these results.

While NR has been previously used to characterise materials containing multiple SCMs,^32^ this did not include samples cured for 28 days, which is the age typically considered by the construction industry in defining cement and concrete performance for design purposes. Also, previous studies have not extended to cementitious blends with substitution levels of blast furnace slag (BFS) beyond 60 wt%, despite existing standards such as EN 197-1^33^ allowing the use of up to 95 wt% BFS in “common cement” blends. The relative assessment of a wide envelope of compositions under laboratory conditions is essential to elucidate the connections between the hydration process and pore structure evolution of cement pastes with high contents of BFS, and its implications for transport properties. This is done with the aim of selecting binder formulation with high SCM contents and with the highest performance potential in highly demanding service environments, including use as waste immobilisation grouts in the nuclear industry. The use of ultra-high volumes of cement replacements (>70 wt%) is considered a key strategy for future decarbonisation of concrete, but the understanding of the microstructural development such cementitious systems is still not complete.

This study investigates cement paste samples with 75 wt% and 90 wt% BFS, coupling non-destructive *in situ* neutron radiography using the NEUTRA neutron radiography beamline at the Swiss Spallation Neutron Source (Paul Scherrer Institute, Villigen, Switzerland),^34^ with synchrotron microtomography using the microtomography beamline XSD-IMG 2-BM at the Advanced Photon Source (Argonne National Laboratory, Chicago IL, USA).^35^ Samples were analysed from 28 to 360 days of age. Microstructural changes occurring in the pore structure during the continuing hydration of the cement and BFS were observed and linked to moisture movement, generating important new insight both into material behaviour, and into the applicability of some of the assumptions that underpin standardised test methods for capillary suction in cements and concretes.

## 2. Experimental methodology

### 2.1 Materials and sample preparation

**Fig. 1:**
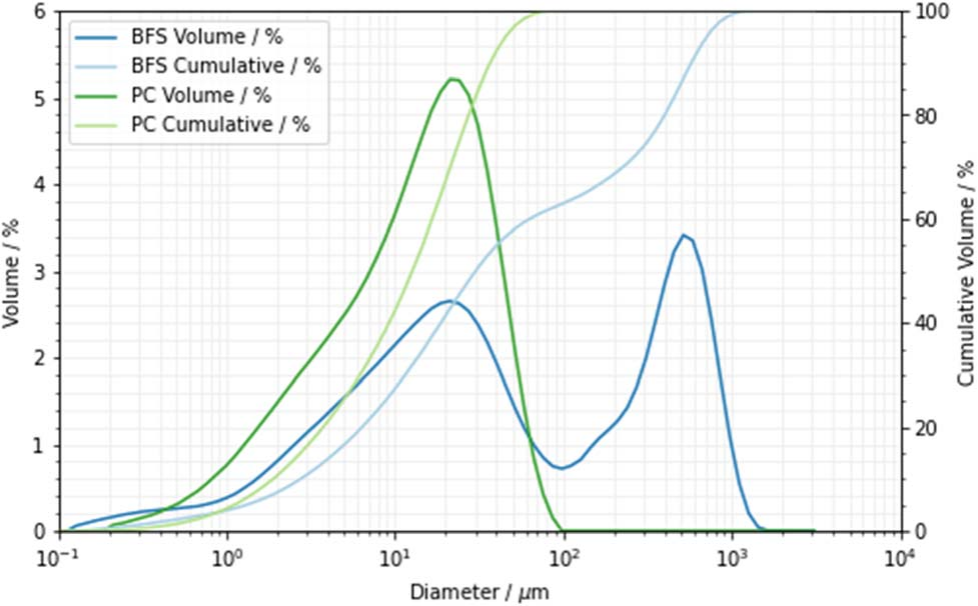
The particle size distributions of the Portland cement (green) and the BFS (blue).

For the NR experiment a total of four specimens were produced from a Hanson Ribblesdale CEM I 52.5N Portland cement (PC) meeting EN 197-1,^33^ blended with a Hanson REGEN BFS at substitution levels of 75 and 90 wt%, falling within the compositional range of the EN 197-1 CEM-III class of blast furnace cements^33^ and also corresponding to some of the mix proportions that are used for the cementation of intermediate level radioactive wastes in the United Kingdom.^36^ X-ray fluorescence data for the PC and BFS materials are shown in Table 1. The particle size distributions of the PC and BFS were determined by laser diffractometry, Fig. 1. The BFS was a blended product supplied to the UK nuclear industry, containing both a fine-ground BFS fraction and a coarse ground BFS fraction (marketed as Calumite), and this product therefore displayed a bimodal particle size distribution. Blaine fineness^37^ values of 376 m^2^ kg^−1^ and 493 m^2^ kg^−1^ were measured for the PC and BFS, respectively.

**Table 1:** X-ray fluorescence spectroscopy data for the PC and BFS materials

Oxide	wt%	
	PC	BFS
CaO	65.4	39.7
SiO_2_	20.0	36.6
Al_2_O_3_	4.6	12.2
SO_3_	3.2	a
Fe_2_O_3_	3.1	0.4
MgO	2.1	8.4
Minor	1.7	2.8

a Not analysed.

The pastes were mechanically mixed at a water to binder (PC + BFS) mass ratio of 0.35, cast into a prismatic block, and cured under sealed conditions for durations of 28 and 90 days before being cut down to a 40 × 40 × 120 mm prism to remove the cast edge and obtain the most homogeneous sample possible for neutron radiography testing. This sizing allowed for the placement of four samples in the field of view while leaving sufficient clearance between each specimen to remove any doubt about sample interference. Prior to testing, samples were preconditioned by heating in a laboratory oven to 105 °C ± 5 °C to a near-constant mass, allowing for the rapid drying of the specimens required due to beamtime scheduling limitations. Though not ideal, this method was compliant with both BS 1881-208,^38^ which is a test method that yields a similar outcome, and complementary pore structure measurements (*e.g.*, ASTM C642-21;^39^ BS 1881-122:2011 ^40^). The method also closely matched previous examples in the literature such as the conditioning approaches of Kanematsu *et al.*,^41^ and Hanžič and Ilić.^42^ Samples were sealed along the four long edges using aluminium tape, which had a low neutron cross section, intending to ensure that uniaxial flow occurred into each specimen as per Lucero *et al.*^43^ Due to the time required to dry specimens prior to the experiment, this was deemed to be the most appropriate method to remove residual moisture from the samples.

For μCT analysis, samples were produced with BFS contents of 75 and 90 wt%, also at a water to binder mass ratio of 0.35, mechanically mixed using a high-shear blade, and cured for 1, 3, 5, 7, and 28 days. Pastes were cast into 2 mm diameter by 5 mm deep holes that had been drilled into a polytetrafluoroethylene block, which was then sealed on both surfaces. Some preferential segregation of the coarsest BFS particles may have occurred during casting, but the use of a high shear regime was intended to minimise this. Once the relevant age had been reached, samples were removed from the mould using a flat plug. Samples of the same cement formulations that had been cured for 360 days were also prepared by sectioning 2 mm-diameter cylinders from large-diameter centrifuge tubes using a slow saw lubricated with mineral oil. Constraints related to beamline scheduling meant that it was not possible to test 90 day cured samples by this technique in this study. Hydration was arrested for all specimens by quenching in isopropyl alcohol for 30 seconds and drying, and this was repeated until a constant mass. This method was selected due to the risk of destruction of very small samples with relatively high temperatures during drying. It was assumed that due to the small cross-sectional area of the samples, differential arrest of hydration across the width was negligible.

### 2.2 Characterisation by X-ray microtomography

The X-ray computerised microtomography instrument at the 2-BM beamline was equipped with a 4-megapixel PCO.edge5.5 16 bit sCMOS camera with a 7.5× magnification Mitutoyo lens and silicon scintillator.^35^ This provided image dimensions of 2016 × 2016 pixels and a nominal pixel size of 0.875 μm per voxel, verified by direct measurement of the sample cross section. As the sample was rotated through 180°, 1801 radiographs were captured using a monochromatic beam with an energy of 19.5 keV. Reconstruction of the microtomographic data was carried out using the TomoPy Python package^44^ and the Fourier grid reconstruction algorithm.^45^ Cubic volumes of interest (VOIs) of 375 μm dimensions were selected at random from within the stack, though due to the presence of large particles (>100 μm), which had a tendency to mask the volume, certain datasets were manually rejected from the analysis where appropriate.

#### 2.2.1. Segmentation and analysis of porosity

**Fig. 2:**
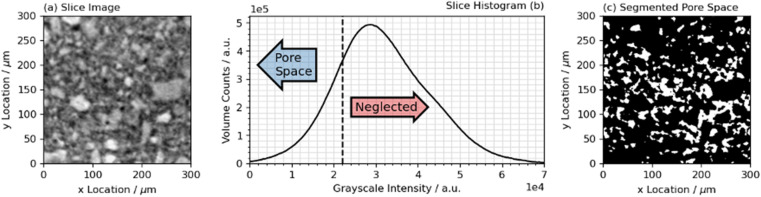
Volume segmentation on the 75 wt% BFS sample at a curing age of 1 day. The reconstructed slice is shown in (a), extracted from the stack with no segmentation. The histogram of the slice shown in (a) is shown in (b), and (c) shows the segmented pore space for the slice in (a). Any material registering above the defined greyscale threshold is neglected in calculations related to pore space.

Images were segmented by applying a global binary threshold operation on each individual slice in the reconstructed stack. The accurate segmentation of the pore space in hardened cement paste is both challenging and potentially subjective, because the fundamental pore size is often below the voxel resolution of μCT and other imaging techniques. Therefore, automated methods have been previously proposed for segmentation, *e.g.* of electron microscopic data, with the aim to minimise human biases.^46^ In this study, segmentation was automated by applying a global binary threshold with the pore space at the maximum rate of increase of histogram counts with grayscale intensity, as shown in Fig. 2. Single voxel pores (0.875 μm) were assumed to lie below the spatial resolution limit of the instrument, were attributed to instrument noise, and were removed. An example of a segmentation calculation is shown in Fig. 2. Minor adjustments were made to account for features such as observable voids (disconnected pore space) present within the larger particles of BFS, where such particles had not been manually removed.

The complexity of the capillary pore volume was characterised by determining the diffusion tortuosity *τ* using the random walk method.^47^ This was calculated by placing 6000 walkers in the segmented pore space and allowing these to simultaneously displace at random (Brownian motion) across 25 000 steps, with the calculation repeated on 5 randomly selected volumes of interest per sample per time point.

The pore size distribution was determined using a local thickness calculation^48^ which was carried out on a single volume, previously segmented. The local thickness method takes a small spherical component and determines all the locations in the volume in which it fits, and this is repeated multiple times with a range of decreasing sizes to the point at which each pixel value is replaced with the diameter of the largest sphere overlapping that region. This provided a measurement of the diameter of each pore in the volume, regardless of the connectivity to the any facet of the VOI. There was no attempt made to quantify the degree of reaction of the BFS or PC material due to the difficulty of accurately segmenting these phases from one another in the reconstructed dataset.

### 2.3 Monitoring water absorption by neutron radiography

**Fig. 3:**
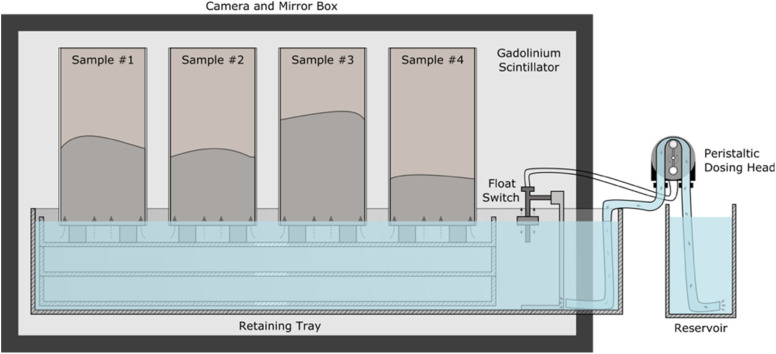
Illustration of the configuration of the experiment in the NEUTRA instrument. The water level was maintained by the peristaltic dosing head activated by the float switch when the water level dropped.

The experiment was conducted using the NEUTRA instrument (Villigen, Switzerland)^34^ with the instrument configured to use sample position three, using a modified ASTM C1585-13 (ref. 8) test configuration for water absorption *in situ* (Fig. 3), where the prismatic sample geometry provided the constant sample cross section required for calibration. Water was initially held in a shallow bath below the base of the samples. A peristaltic dosing head which was connected to a reservoir and float switch was triggered to fill the bath when the experiment was started (to enable remote triggering after the beam had been activated, minimising any loss of information due to the time required to open shutters and begin collecting radiographs), and the float switch was subsequently used to maintain contact between the water and sample base by maintaining a near-constant water level throughout the experiment.

The NEUTRA beamline was equipped with an Andor NEO sCMOS detector and a gadolinium scintillator attached to a mirror box. This provided a nominal pixel size of 10 μm, verified by direct image measurement. Scattering corrections were carried out by placing a masked polymer block in front of the experiment and subtracting the resulting image. Four samples were placed in front of the scintillator, with the limited number of samples resulting from restrictions in scheduling and the instrument field of view. Data were captured for 24 hours during capillary moisture uptake, producing a series of 288 time-resolved two-dimensional images. The image capture time was calculated, and each image was corrected for dark current and open beam artefacts, though it was found that entire elimination was not possible due to flux variations between capture of the dry sample and open beam images which indicated as variations in grayscale intensity across the imaged cross section. Scattering of neutrons from the mirror was corrected for by subtraction of the black body image prior to further processing.

#### 2.3.1. Location of the wetting front

The location of the wetting front was determined by taking each vertical line in the *x* direction as a linear signal. This was smoothed by convolving a Hanning window across the signal and calculating the maximum rate of change in grayscale intensity with respect to the *y* axis. This was carried out at every point in the *x* axis, and the mean of the maximum rate of change was determined in every *x* position across the sample surface. The location of the wetting front was then determined by taking the nominal pixel size of the detector and assuming a perfectly parallel beam. This had the effect of smoothing out variations in the shape of the wetting front, though provided a solution which allowed for repeatability in all samples.

#### 2.3.2. Dry specimen contribution

Upon the initial completion of data processing and subtraction of the contribution of the dry sample, a minor degree of incomplete drying was observed in the specimens with a high degree of hydration (cured for 90 days), which occurred due to the ability of the refined pore structure in these samples to both retain moisture, and to prevent residual moisture from redistributing once drying was complete. This caused an artefact where an excessive amount of moisture had been subtracted below the wetting front. In this case, the analysis was written to assume that there was water present in the sample only below the wetting front. To do this, the residual moisture in the specimen was removed by randomly selecting values from the top 2 cm of the dry specimen and replacing the matrix values in the dry specimen below the wetting front (determined from the wet image) with these sampled values. By doing so, excessive subtraction of values below the wetting front was prevented, and the final image was able to accurately depict the region without distortion or interference.

#### 2.3.3. Moisture content calibration

To calibrate the moisture content present in each sample to a known value, images of a dry specimen were captured showing blocks of a known water path length (step wedges). This is a commonly applied calibration method, and though the application of modified Beer–Lambert law can also potentially yield a moisture content result, the method applied requires no assumptions regarding absorption coefficients and eliminates any potential underestimation of the absorbed content, an effect previously observed by Kang *et al.*^49^

**Fig. 4:**
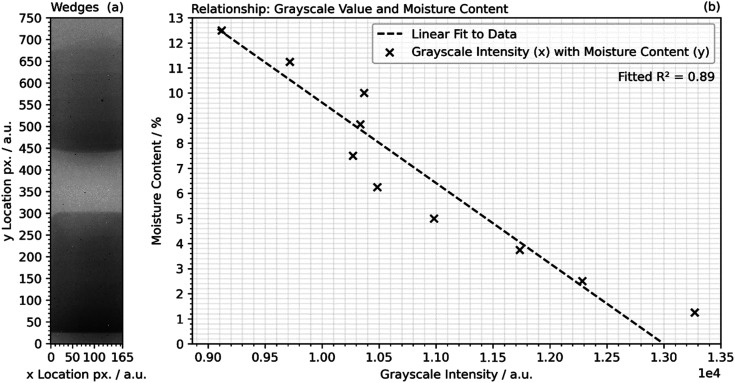
(a) A radiograph of a dry sample with step wedges superimposed after processing for corrections as described in the text, and (b) the relationship between the greyscale step wedge intensity and the resolved moisture content.

Therefore, blocks representing specific moisture contents were placed in front of each dry sample, as shown in Fig. 4(a) and following eqn (1), where *t*_w_ is the thickness of each wedge and *t*_s_ is the thickness of the sample (circa. 40 mm). The mean grayscale value at each step, where the moisture content was known, was calculated and fitted to a linear function by least squares regression fitting, Fig. 4(b). It was assumed that the moisture content in each sample was satisfactorily represented by the same calibration function. The slight deviation from a linear relationship resulted from a combination of parabolic flux through the beam cross-section from imperfect collimation,^34^ and progressive burn-in of the scintillator which could not be entirely accounted for. Fitting yielded an *R*^2^ value of 0.89 which was considered acceptable for this analysis.1
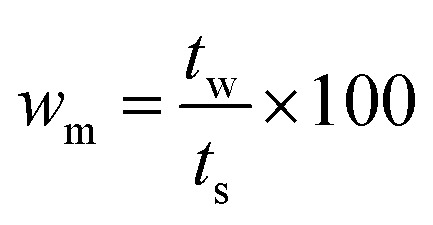


## 3. Results and discussion

### 3.1 Formation of the pore structure

**Fig. 5:**
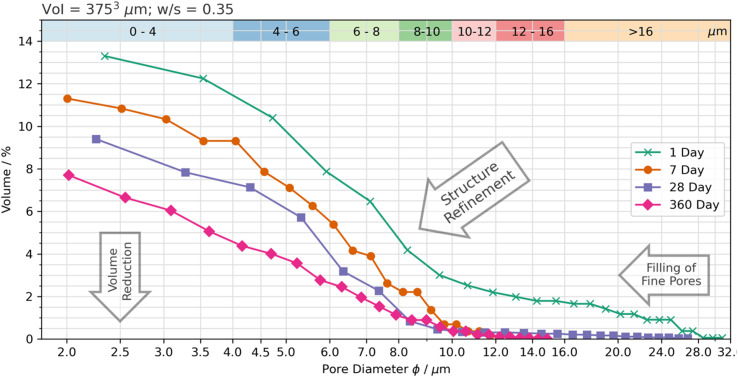
Pore size distribution for the 75 wt% BFS sample, as a function of curing time.

**Fig. 6:**
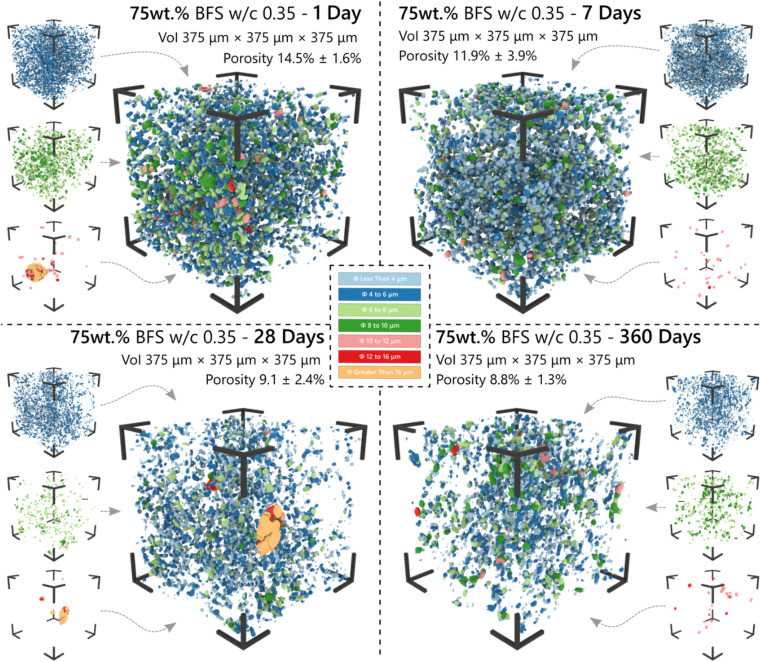
The 3-dimensional pore network distribution of the 75 wt% BFS sample as determined by μCT analysis of a volume of interest (VoI) as a function of the curing time. Pore size ranges are distinguished by colour, as indicated.

**Fig. 7:**
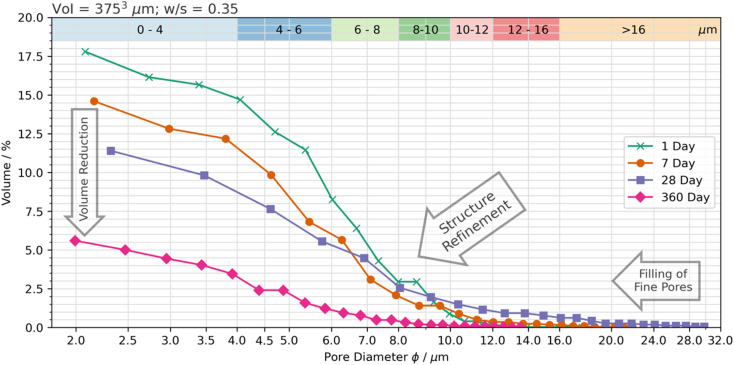
Pore size distribution for the 90 wt% BFS sample, as a function of the curing time.

**Fig. 8:**
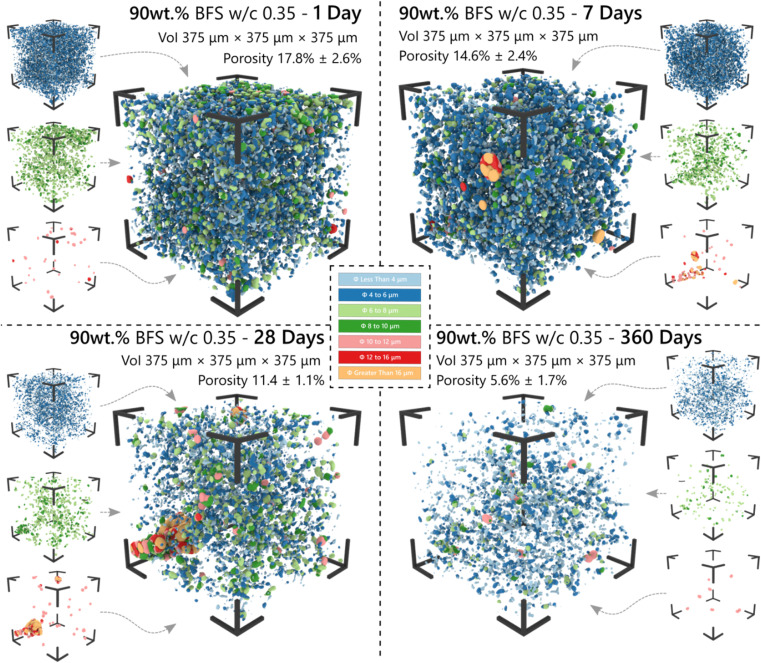
The 3-dimensional pore network distribution of the 90 wt% BFS sample as determined by μCT analysis of a volume of interest (VoI) as a function of the curing time. Pore size ranges are distinguished by colour, as indicated.

The three-dimensional distribution and sizes of capillary pores within the VOI is shown in Fig. 5 and 6 for the 75 wt% BFS blend, and Fig. 7 and 8 for the 90 wt% BFS blend, respectively. The volumes shown are typical of the reconstructed volume for each composition and curing age. The capillary pores that can be imaged by this technique are in the larger end of the overall capillary pore range, with diameters between 2 μm and 31 μm. Large BFS particles contained entrapped bubbles and were manually removed from each VOI. Pores greater than 16 μm were assumed to have resulted from voids incorporated in the wet paste from incomplete compaction.

The total porosity was determined by voxel counting and returned values are reported in Table 2. Porosity values range between 5.6 and 17.8 vol%, significantly reducing as the samples aged, independently of the BFS content in the hardened cement paste. This is consistent with results reported for BFS blended cements,^50^ where the effectiveness of BFS reaction in inducing pore refinement has been demonstrated. A slightly higher porosity was identified at early curing ages (up to 28 d; Fig. 6) when using 90 wt% BFS compared with 75 wt% BFS, which is expected due to the combined effect of delayed reaction of BFS compared with PC at such high replacement levels,^51^ and also the decrease in PC hydration at high BFS replacement levels.^50^ However, at an advanced curing age (360 days), the total porosity of the 90 wt% BFS containing cement was significantly lower (5.6%) than that of the 75 wt% BFS blend (8.8 wt%). These results are attributed to the marked pore refinement taking place with the increase of BFS content in the hardened paste at the replacement levels evaluated in this study (Fig. 5 and 7). This demonstrates that the reaction of BFS continues at extended curing times (360 days), and the precipitation of reaction products is sufficient to continue inducing a significant pore structure refinement.

**Table 2:** Total porosity (vol%) of BFS blended cement pastes, determined by μCT, as a function of curing duration

Curing age (days)	Porosity (vol%)	
	75 wt% BFS	90 wt% BFS
1	14.5 ± 1.6	17.8 ± 2.6
7	11.9 ± 3.9	14.6 ± 2.4
28	9.1 ± 2.4	11.4 ± 1.1
360	8.8 ± 1.3	5.6 ± 1.7

Recent results have reported a porosity of 20.7% in a similar binder composition (60 wt% BFS with water/binder = 0.30, cured for 7 days), determined in three dimensions using laser scanning confocal microscopy.^52^ Those results were captured at a higher resolution of 0.156 μm (*X*,*Y* axis)/0.534 μm (*Z* axis) and hence incorporated pores of a significantly lower diameter than was achieved in the current study, which explains the higher porosity measured. Promentilla *et al.*^53^ reported total porosities between 5% and 21% using synchrotron μCT data studying samples of pure PC between 2 and 28 days of curing with a reported voxel size of 0.5 μm, which closely matches the range presented here although with a significantly different sample composition. Based on these results, we are confident of the accuracy of our reported values given the limitations of the instrument used to acquire these data, and of the segmentation method applied.

#### 3.1.1. Determination of tortuosity

**Fig. 9:**
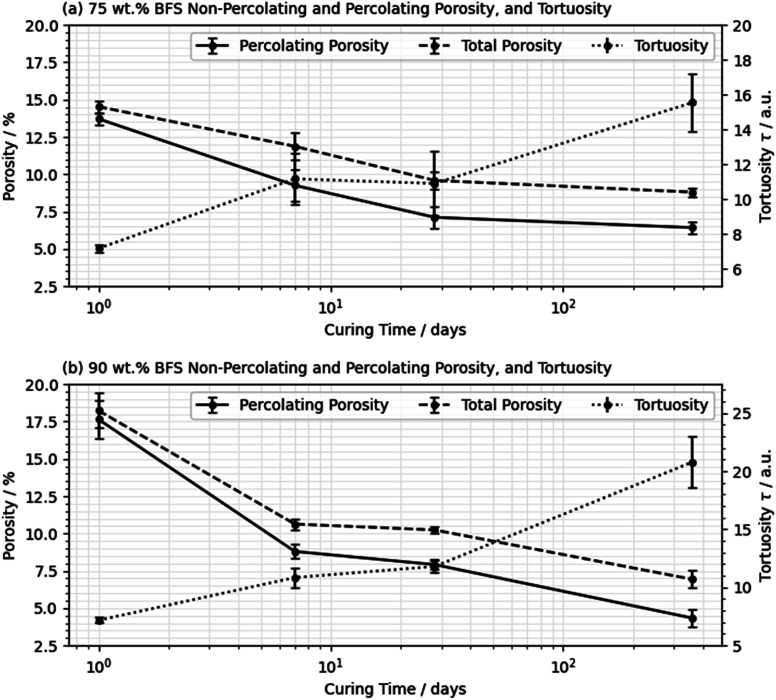
The percolating and non-percolating porosity (left axis) and tortuosity (right axis) of the 75 wt% BFS (a) and 90 wt% BFS (b) samples, cured for 1, 7, 28, and 360 days. Error bars are standard errors determined from repeat analyses on five randomly selected volumes of interest.

Analysis of the tortuosity was performed initially on the volumes presented in Fig. 6 and 8, with a further four volumes randomly selected from the stack at each age. Fig. 9 provides the total and percolating porosity, and the tortuosity of the 75 wt% BFS blend in (a) and the 90 wt% BFS blend in (b). The complete closure of some of the pores was indicated by the divergence between the volume of percolating and non-percolating porosity, and the decrease in interconnectivity was most rapid during the first 7 days of curing where the hydration reactions occurred at the most rapid rate.^51^ The tortuosity values presented in Fig. 9 are also broadly consistent with the corresponding values determined for Portland cement hydration at early age (*e.g.* a tortuosity of 4.0 after 14 hours of hydration) in ref. 2.

As the PC and BFS within each sample hydrated, the path complexity increased due to the in-filling of hydrates into the empty pore space, reducing the extent to which a single walker was able to displace before colliding with the pore wall.^2^ This translated into a reduction in the mean rate of walker displacement and an increase in the tortuosity. It was noted that tortuosity values for similar materials presented in the literature varied greatly based not only on the composition but also the specifics of the methodology applied. Reported tortuosity values have been as low as between 2 and 3 for empirical relationships formed based on diffusion coefficients in hydrated BFS blended cement pastes,^54^ to several hundred for each axis in highly anisotropic materials.^24^ Results have been reported as high as between 900 and 1000 when measuring the electrical conductance.^55^ Based on the other data available, it was evident from our results and the broader literature^56,57^ that the tortuosity was not agnostic to the applied analytical method, and thus it is unlikely that results obtained across different research studies are truly comparable to one another.

Using the method applied here, curing resulted in an increase in the tortuosity, and this occurred to a greater extent in the 90 wt% BFS sample than at 75 wt% replacement, after 360 days of curing, consistent with the reduced total porosity identified in the 90 wt% BFS sample (Fig. 8). From these results it is possible to hypothesise that a decrease in absorption will follow in the 90 wt% BFS blend at extended degrees of hydration, in the neutron radiography experiments to be presented below. It may also be expected that alongside a decrease in absorption, that the moisture content below the wetting front will also be reduced.

### 3.2 Water absorption by *in situ* neutron radiographic imaging

To monitor the effect of cement replacement and hydration progress, neutron radiographic imaging was carried out across the first 24 hours of absorption, for samples comprised of 75 and 90 wt% BFS blended cements that had been cured for 28 and 90 days. Alongside being sensitive to the moisture content distribution through the sample cross section, this experiment allows for the detection of the precise rate and depth of absorption through the sample to be evaluated. By comparison to standardised test methods which are purely gravimetric, this approach removes the requirement for simplifying assumptions that relate the change in sample mass to the infiltration depth of the wetting front.

#### 3.2.1. Distribution of moisture and the wetting front

**Fig. 10:**
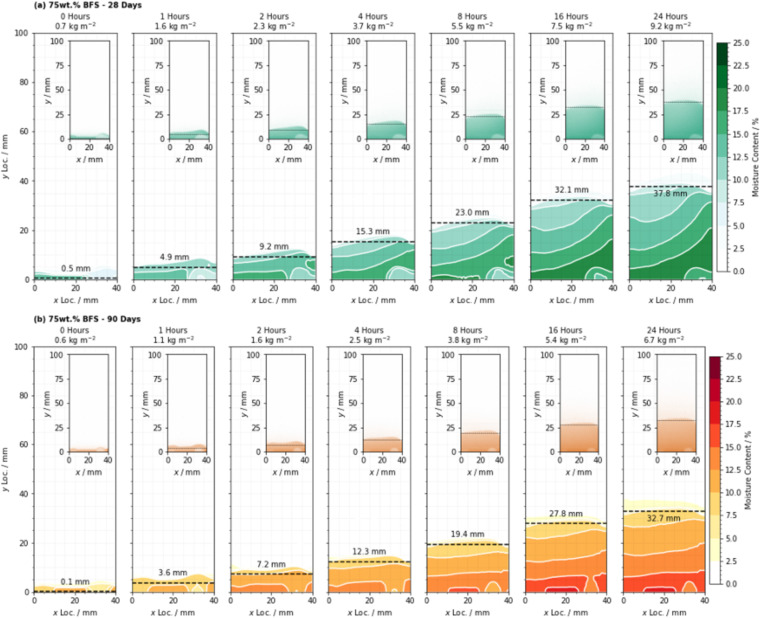
Distribution of moisture during the capillary absorption tests as determined by quantitative neutron radiography, for the 75 wt% BFS sample, cured for (a) 28 days, and (b) 90 days. Each image is annotated with the total water uptake per unit cross-sectional area corresponding to that time point, and the position of the wetting front as defined by summing the data in the *x* direction and then differentiating with respect to *y*. Each image corresponds to a 5 minute exposure duration, commencing at the time indicated.

**Fig. 11:**
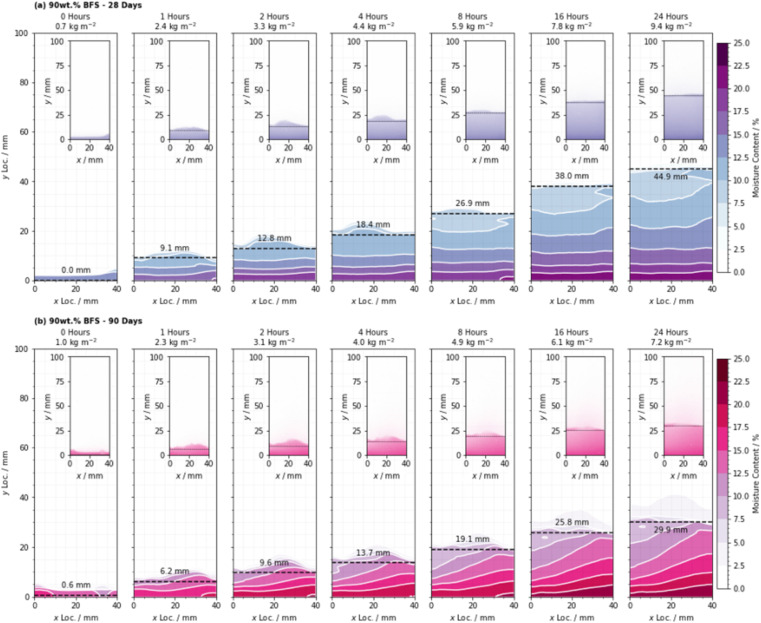
Distribution of moisture during the capillary absorption tests as determined by quantitative neutron radiography, for the 90 wt% BFS sample, cured for (a) 28 days, and (b) 90 days. Each image is annotated with the total water uptake per unit cross-sectional area corresponding to that time point, and the position of the wetting front as defined by summing the data in the *x* direction and then differentiating with respect to *y*. Each image corresponds to a 5 minute exposure duration, commencing at the time indicated.

The quantified distribution of moisture content for all samples tested, across the 24 hours of the experiment, is shown in Fig. 10 for the 75 wt% BFS specimens cured for 28 and 90 days. Fig. 11 similarly shows the distribution of moisture in the 90 wt% BFS specimens. Water absorption began immediately on contact of the sample with water, prior to the time at which the capture of the first image was completed, and some minor absorption occurred at the point of the sample supports due to moisture that remained in the apparatus from previous test runs.

Analysis of the distribution of moisture in the specimens indicated that saturation below the wetting front did not occur instantaneously on contact with the proceeding front. The shape of the wetting front in each sample was curved with a maximum rate of ingress near the sample centreline and minima at each edge, which resulted from resistance to moisture transport at the interface between the concrete and the sealing aluminium, consistent with the results presented by Alderete *et al.*^32^

The absorption of water into hardened cementitious materials and concretes is traditionally quantified either gravimetrically as per the ASTM C1585 (ref. 58) method or by volume as per the BS 1881-208 “Initial surface absorption test” (ISAT).^38^ However, both of those approaches provide only a low-resolution mass-derived outcome which lacks any detail regarding the actual depth of moisture penetration. This is important for studies into, for example, corrosion, where the positioning of rebar and the ability for moisture to contact it is significant, or freeze-thaw cycling where the overall depth to which water is absorbed significantly affects fracturing or spallation of cover concrete. Without further context this is a potentially arbitrary parameter.

In order to convert the data into a useful quantity, it is possible to take the results from the certain tests (*e.g.*, the ISAT) and derive a full infiltration depth measurement, for example using the method derived by Claisse^59^ which gives eqn (2). Here, *F*_v_ is the observed flux (m^3^ s^−1^), *s* is the surface tension of water, *K* is the intrinsic permeability (measured or assumed), *A* is the cross section area of the sample, *e* is the viscosity of water, *r* is the radius of the largest capillary, and *X* is the infiltration depth. This approach assumes a sharp wetting front with an entirely uniform distribution of moisture below, and an immediate change in moisture content at the leading edge.2
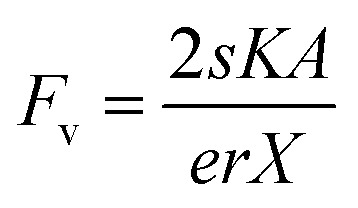


In this study it was not possible to identify a sharp wetting front with an immediate fall-off in moisture content, and alongside the non-uniform moisture distribution through the sample depth and across the width, this indicated that such assumptions may not be valid for cementitious materials with high degrees of SCM substitution. This raises the need to carefully reconsider the validity of information resulting from standard tests that are derived from these assumptions, especially when converting laboratory test results into performance predictions for cementitious materials in service based on secondary relationships.

#### 3.2.2. Direct measurement and quantification of the absorption process

**Fig. 12:**
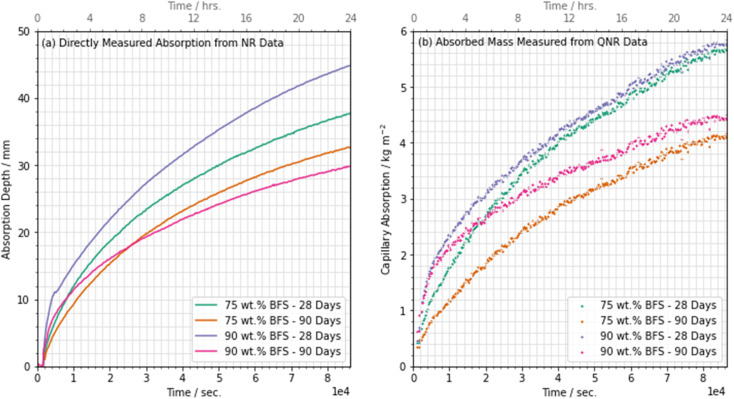
Absorption of moisture into each sample tested, as labelled, represented as: (a) the absolute absorption directly measured from the NR data, and (b) the quantified mass of absorbed water, plotted against linear time showing a parabolic absorption curve.

Having quantified the moisture content in each location in the cross section, it is possible to both determine the mass of water absorbed (kg m^−2^) by each sample and to determine the actual depth to which moisture has been absorbed (mm). The absorption of water into each sample followed a smooth monotonic curve, where a rapid initial absorption process progressively reduced in rate, as shown in Fig. 12(a) which shows the absorption depth (from NR) and Fig. 12(b) which shows the mass absorbed (from QNR) for each sample, as labelled.

Water was observed to reach a maximum infiltration depth of 44.9 mm after 24 hours in the 90 wt% BFS sample that had been cured for 28 days; the relatively high absorption in this sample was a result of the slower hydration of the BFS at such a high degree of substitution for PC.^51^ This is also consistent with the porosimetry results presented in Fig. 5 and 7, where the 90 wt% BFS sample was more porous than 75 wt% BFS at early age, but much less porous at later age.

However, the measured infiltration depth and the absorbed mass did not follow the same trend: the depth measurements suggested that the 90 wt% BFS sample outperformed the 75 wt% BFS sample when both had been cured for 90 days, in agreement with the tortuosity and porosity results obtained from the microtomography data in Section 3.1, where Fig. 9 shows that the 90% BFS sample developed a pore network that was more tortuous and less percolating. However, the absorbed mass of water was greater in the 90% than the 75% BFS sample after 90 days of curing (and the two formulations showed similar mass uptake values when cured for 28 days). The main difference in the 90 day cured samples is seen in Fig. 12b in the first hours of water uptake, corresponding to a pore fraction which can rapidly absorb a large amount of water without a high measured absorption depth. It is possible that at this age, the significant fraction of pores >2 μm that were observed at early age in Fig. 7 have moved into a size regime which is too small to view by X-ray microtomography here but is still active in capillary uptake of moisture. This may have then led to the apparent mismatch between measured mass uptake (which would conventionally be used to quantify the adsorption) and the actual measured water absorption depth when comparing the 75 wt% and 90 wt% BFS samples. It is therefore possible that the typical infiltration equation (*e.g.*, ref. 8) may lead to misleading results in samples which are highly substituted with SCMs, and/or when testing large cementitious paste samples such as those used here (and widely in the nuclear industry) due to the challenges associated with casting a sample with truly homogeneous pore structure in the absence of any aggregate.

#### 3.2.3. Single term parameters for absorption

The ability for a specimen to absorb water through capillary action has been previously linked to the durability of a specimen, for example the carbonation^60^ or chloride permeability.^61^ In order to use a measurement of absorption to produce meaningful relationships, this must be defined as a single-term quantity. This has often been done by assuming a functional form with a square-root time dependency, and applying a least-squares regression fit to obtain a capillarity coefficient which is used as a proxy metric to predict the durability of the material. However, techniques are now advancing due to recognition of the significant non-linearity that is observed during the water absorption process in cementitious materials. While measurements of sample deformation have recently suggested that non-linear absorption begins very early on in the absorption process,^62^ this becomes especially prevalent in the absorption data beyond a duration of between 6 and 24 hours, and the numerous testing standards deal with this in various ways. For example, the ASTM C1585 standard^8^ divides absorption into two periods, the first up to 6 hours and the second beyond 24 hours, while EN 13057:2002 neglects one or the other portion of the curve depending on whether or not full saturation of the sample is reached within 24 hours.^63^ BS 1881-208 (ref. 59) neglects the effect entirely. Recent results have suggested that this effect is most likely caused by the tendency for calcium silicate hydrate-like products (C-S-H, or C-[A]-S-H if some aluminium substitution is considered) to expand on contact with water, constricting the pore structure and limiting the ability of water to migrate through capillary action.^64^

**Fig. 13:**
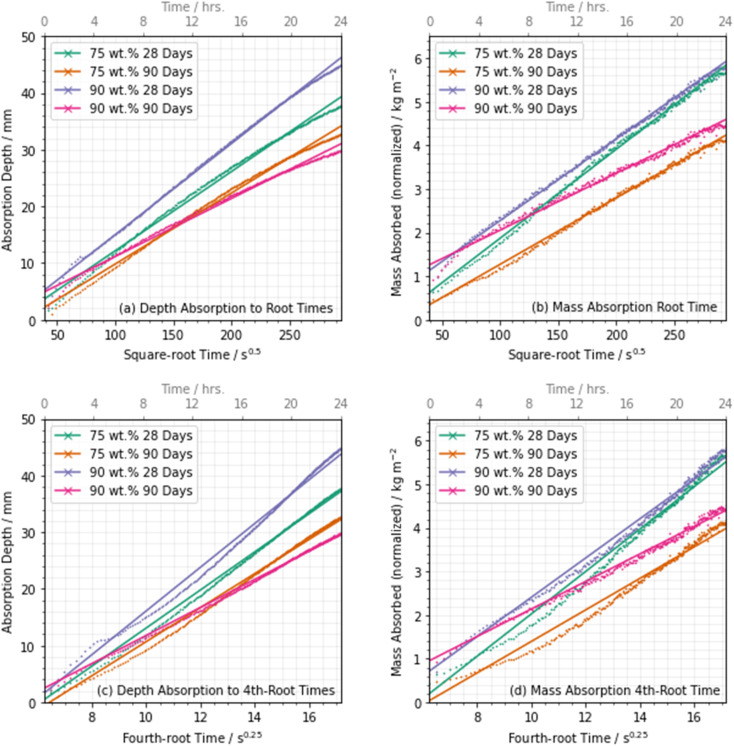
Absorption data plotted as a function of (a,b) the square root, and (c,d) the fourth root of time, with the measured depth (a,d) and the measured mass (b,d) shown. Straight lines show the linear fit to each, with fit parameters given in Table 2.

Due to this non-linear absorption, the single-parameter coefficient of water sorptivity fitted to a time^1/2^ function is becoming recognised as unable to provide an accurate description of the secondary absorption period, especially in a research-focussed setting where high accuracy is desirable. Discussion in the literature has suggested that fitting to the fourth-root of time may provide a more appropriate result.^65^ So in order to assess the effect of non-linear absorption during this experiment, the data were fitted to both the square-root and fourth-root of time using least squares regression fitting. This was carried out for both the directly measured (NR) absorption results in Fig. 13a (square-root) and Fig. 13c (fourth-root), and the quantitative neutron radiography data (QNR) in Fig. 13b (square-root) and Fig. 13d (fourth-root). Goodness-of-fit parameters, and sorptivity or absorption rate values, are provided in Table 3.

**Table 3:** Absorption and sorptivity values from square root and fourth root fits shown in Fig. 13, and corresponding goodness of fit parameters

Sample		Square-root time			Fourth-root time		
		Sorptivity (kg m^−2^ s^−0.5^)	Absorption rate (mm s^−0.5^)	*R* ^2^ coeff.	Sorptivity (kg m^−2^ s^−0.25^)	Absorption rate (mm s^−0.25^)	*R* ^2^ coeff.
75 wt% BFS	Direct (NR)	—	1.36	0.998	—	32.57	0.984
28 day	Mass (QNR)	1.25	—	0.998	29.95	—	0.986
75 wt% BFS	Direct (NR)	—	1.23	0.998	—	29.35	0.982
90 day	Mass (QNR)	0.84	—	0.997	20.01	—	0.984
90 wt% BFS	Direct (NR)	—	1.59	0.999	—	38.06	0.986
28 day	Mass (QNR)	1.82	—	0.997	43.89	—	0.989
90 wt% BFS	Direct (NR)	—	1.00	0.996	—	24.22	0.990
90 day	Mass (QNR)	0.88	—	0.991	21.31	—	0.992

These results showed that a very slight decrease in the quality of fit (*R*^2^ coefficient) occurred when changing from a square-root of time to a fourth-root of time fit, for all specimens tested, indicating that this proposed functional form did not necessarily provide a significant advance over the established method for the data sets presented here. However, this may also be an effect of the preconditioning method, and processing of the sample at an elevated temperature has been shown to potentially result in an increase in linearity with square root-time by Castro *et al.*^66^ Therefore, with only a small variation in the *R*^2^ coefficient, it is challenging to confidently draw a conclusion regarding this effect, and further testing with samples processed under different conditions and for an increased duration is therefore necessary to fully investigate this. Fig. 13d in particular shows that the data are visibly non-linear throughout the time period samples when plotted against the fourth-root of time. Deviations from linearity in the square-root time relationship are seen in the first few hours of absorption. It was apparent that sufficient secondary absorption to restrict the progress of the wetting front did not occur within the first 24 hours of the absorption process in the case of these samples which caused absorption to remain linear when fitted to square-root time functions.

#### 3.2.4. Influence of the slag content and curing age

Analysis of these results indicated that the initial rate of moisture absorption into the 90 wt% BFS blended cement decreased significantly between 28 and 90 days (−0.59 mm s^0.5^), and this occurred to a far more significant extent than for that of the 75 wt% BFS material where the change was not so marked (−0.13 mm s^0.5^). This was found to be consistent with the changes in pore size distribution (Fig. 5 and 7), where the decrease in the total pore volume in the 75 wt% BFS material beyond 28 days was reduced, relative to that of the 90 wt% BFS blend.

This may have resulted from one of several effects. In the case of the addition of finely divided powders into the cement, the filler effect has been shown provide additional sites for nucleation, which triggers the precipitation of additional volumes of C-(A)-S-H,^67,68^ and it thus follows that this C-(A)-S-H enters and fills the pore space. Additionally, fine particles may pack the interparticle spacing with fine particles, inhibiting absorption while also increasing the compressive strength.^69^ The trend observed would be inconsistent with the slag having a packing effect, because the reduction in absorption rate was not decreased at very high slag content where the fine particles in the slag would act as the filler. It is therefore more likely that this resulted from the formation of additional volumes of C-(A)-S-H, although it was not apparent as to whether this had occurred from the hydration of the slag or nucleation effects.

## 4. Conclusions and remarks

In this paper, the relationship between the formation of the pore structure and the capillary absorption in highly substituted Portland cement BFS blends was characterised. Microtomographic imaging data were collected on a number of samples at different curing durations and indicated a trend towards pore refinement alongside a decrease in the overall pore volume. This volume of percolated porosity, contributing to the accessible pore space also decreased. The reduction in porosity and increase in tortuosity occurred to a greater extent in the 90 wt% BFS material than at 75 wt% BFS, and by 360 days the 90 wt% composition had the potential to outperform the 75 wt% material in terms of resistance to capillary moisture movement.

However, it is plausible also that pores above 2 μm in diameter were refined into a size regime that was undetectable by the X-ray microtomography technique, and though smaller, these pores continued to actively participate in the absorption process. These smaller yet actively participating pores appear to have potentially influenced capillary moisture absorption, accounting for the observed disparity between theoretically estimated mass uptake traditionally indicative of adsorption depth and the empirically ascertained water absorption depth presented in this paper. Considering this, it is important to also acknowledge that the two techniques applied in this paper were capable of covering fundamentally different length-scales. While microtomography was able to capture the geometry of the pore structure on a length-scale of microns to tens-of-microns while neglecting the absorption dynamics, neutron radiography captured bulk movement of water and the effect of the moisture content, though was incapable of probing the geometry of the pore space. The congruence observed between the microscopic pore geometry (tortuosity; pore size distribution; connectivity) and the macroscopic absorption data suggests that pore structure properties are interconnected across these length-scales, though the contribution of smaller capillary active pores below the length scale achievable by microtomography does require further investigation. The results presented here therefore evidence how challenging it is to characterise the wide spectrum of pore sizes and absorption phenomena using non-destructive analytical methods in isolation.

By monitoring absorption using neutron radiographic imaging it was not possible to observe any non-linearity in the absorption curve within 24 hours, and it is suggested that the literature could benefit from analysis using further testing using techniques which are able to map the swelling on the surface of a sample at a very high resolution. Our results also indicated that the assumptions associated with gravimetric modelling of absorption using standardised methods do not necessarily hold true for cementitious materials with very high degrees of substitution, and that this may have led to misleading results using the “sharp front” model. Further testing ideally requires investigation with a wider envelope of sample compositions and ages to validate this observation. It is also possible to suggest from these results that testing at 28 days is not appropriate for materials which contain such a high quantity of BFS due to the frequently observed low hydration rate of SCMs. It is therefore not possible to predict the long-term performance of these hardened cement pastes from results obtained after only 28 days of curing.

## Data availability

The data associated with this paper are openly available from the University of Leeds Data Repository, at https://doi.org.10.5518/1471

## Author contributions

J. E. Vigor – methodology; investigation; data curation; formal analysis; writing – original draft; writing – review and editing; visualization. D. P. Prentice – investigation; writing – review and editing. X. Xiao – investigation; writing – review and editing. S. A. Bernal – conceptualization; formal analysis; supervision; writing – review and editing; funding acquisition. J. L. Provis – conceptualization; methodology; investigation; formal analysis; writing – review and editing; supervision; project administration; funding acquisition.

## Conflicts of interest

The authors declare that they have no known competing financial interests or personal relationships that could have appeared to influence the work reported in this paper.

## Supplementary Material
